# Efficacy and safety of Velmanase alfa in the treatment of patients with alpha-mannosidosis: results from the core and extension phase analysis of a phase III multicentre, double-blind, randomised, placebo-controlled trial

**DOI:** 10.1007/s10545-018-0185-0

**Published:** 2018-05-30

**Authors:** Line Borgwardt, Nathalie Guffon, Yasmina Amraoui, Christine I. Dali, Linda De Meirleir, Mercedes Gil-Campos, Bénédicte Heron, Silvia Geraci, Diego Ardigò, Federica Cattaneo, Jens Fogh, J. M. Hannerieke Van den Hout, Michael Beck, Simon A. Jones, Anna Tylki-Szymanska, Ulla Haugsted, Allan M. Lund

**Affiliations:** 1 Department of Paediatrics and Adolescent Medicine, Centre for Inherited Metabolic Diseases, Copenhagen, Denmark; 2 Center for Genomic Medicine, Copenhagen University Hospital, Rigshospitalet, Copenhagen, Denmark; 3 Centre de Référence des Maladies Héréditaires du Métabolisme, Hôpital Femme Mère Enfant, Lyon, France; 4 Center for Pediatric and Adolescent Medicine, University Medical Center Mainz, Villa Metabolica, Mainz, Germany; 5 Paediatric Neurology and Metabolism, Universitair Ziekenhuis, Brussel, Belgium; 6 Unidad de Metabolismo e Investigación Pediátrica, Hospital Universitario Reina Sofía, IMIBIC, Universidad de Córdoba, CIBERObn, Córdoba, Spain; 7 Department of Pediatric Neurology, Reference Center for Lysosomal Diseases, Trousseau Hospital, APHP, and GRC ConCer-LD, Sorbonne Universities, UPMC University 06, Paris, France; 8 Chiesi Farmaceutici S.p.A, Parma, Italy; 9 Zymenex A/S, Hillerød, Denmark; 10 Center for Lysosomal and Metabolic Diseases (Department of Pediatrics), Erasmus MC University Medical Center – Sophia Children’s Hospital, Rotterdam, The Netherlands; 11 Institute of Human Genetics, University Medical Center, Mainz, Germany; 12 Manchester Centre for Genomic Medicine, Central Manchester University Hospitals NHS Foundation Trust, Manchester, UK; 13 Department of Paediatric, Nutrition and Metabolic Diseases, The Children’s Memorial Health Institute, Warsaw, Poland; 14 Department of Occupational Therapy and Physiotherapy, Copenhagen University Hospital, Rigshospitalet, Copenhagen, Denmark; 15 Department of Clinical Genetics, Centre for Inherited Metabolic Diseases, Copenhagen University Hospital, Rigshospitalet, Copenhagen, Denmark

## Abstract

**Introduction:**

This phase III, double-blind, randomised, placebo-controlled trial (and extension phase) was designed to assess the efficacy and safety of velmanase alfa (VA) in alpha-mannosidosis (AM) patients.

**Methods:**

Twenty-five patients were randomised to weekly 1 mg/kg VA or placebo for 52 weeks. At study conclusion, placebo patients switched to VA; 23 patients continued receiving VA in compassionate-use/follow-on studies and were evaluated in the extension phase [last observation (LO)]. Co-primary endpoints were changes in serum oligosaccharide (S-oligo) and in the 3-min stair-climb test (3MSCT).

**Results:**

Mean relative change in S-oligo in the VA arm was −77.6% [95% confidence interval (CI) −81.6 to −72.8] at week 52 and −62.9% (95% CI −85.8 to −40.0) at LO; mean relative change in the placebo arm was −24.1% (95% CI −40.3 to −3.6) at week 52 and −55.7% (95% CI −76.4 to −34.9) at LO after switch to active treatment. Mean relative change in 3MSCT at week 52 was −1.1% (95% CI −9.0 to 7.6) and − % (95% CI −13.4 to 6.5) for VA and placebo, respectively. At LO, the mean relative change was 3.9% (95% CI −5.5 to 13.2) in the VA arm and 9.0% (95% CI −10.3 to 28.3) in placebo patients after switch to active treatment. Similar improvement pattern was observed in secondary parameters. A post hoc analysis investigated whether some factors at baseline could account for treatment outcome; none of those factors were predictive of the response to VA, besides age.

**Conclusions:**

These findings support the utility of VA for the treatment of AM, with more evident benefit over time and when treatment is started in the paediatric age.

**Electronic supplementary material:**

The online version of this article (10.1007/s10545-018-0185-0) contains supplementary material, which is available to authorized users.

## Introduction

Alpha-mannosidosis (AM) is a rare autosomal recessive lysosomal storage disorder with a prevalence estimated at 1–2:1,000,000 live births (Meikle et al. 1999; Meikle et al. 2004). AM is characterised by a deficiency of the lysosomal enzyme, alpha-mannosidase, caused by pathogenic sequence variants in the *MAN2B1* gene. This deficiency leads to accumulation of mannose-rich oligosaccharides, causing impaired cellular function and apoptosis, which conversely leads to significant and diverse adverse clinical manifestations (Beck et al. 2013; Borgwardt et al. 2015). Currently, the only treatment option for AM is allogeneic haematopoietic stem cell transplantation (HSCT). However, not all patients are eligible for HSCT or can be matched with compatible donors; HSCT results are variable, and treatment carries mortality risks (Mynarek et al. 2012; Danielsen et al. 2013).

Velmanase alfa is a recombinant human alpha-mannosidase in development for weekly intravenously administered (IV) enzyme replacement therapy (ERT) for AM (Borgwardt et al. 2013). Evaluation of velmanase alfa (VA) in phases I (rhLAMAN-02; NCT01268358) and II (rhLAMAN-03/rhLAMAN-04; NCT01285700/ NCT01681940) clinical studies showed a significant reduction in serum oligosaccharides at 18 months and significant improvements in key clinical parameters of the disease (Borgwardt et al. 2014).

This paper reports results of the phase III clinical trial (rhLAMAN-05; NCT01681953) designed to evaluate the efficacy and safety of 1 mg/kg weekly IV VA treatment compared with placebo at 52 weeks in patients with AM. Treatment dose was previously selected on the basis of the phase I/II dose of 25 U/kg (Borgwardt et al. 2014), which corresponds to 0.8 mg/kg, rounded to 1 mg/kg for calculation convenience. Additional data from an extension phase assessing the long-term effects of VA and describing the effects of switching from placebo to active treatment are also presented.

## Methods

### Study design

This Phase III, international, multicentre, double-blind, randomised, placebo-controlled, parallel-group trial was designed to investigate the efficacy and safety of VA treatment of patients with AM. Patients who received VA during were able to continue receiving treatment in a compassionate-use (CU) programme/follow-on trial [rhLAMAN-07 (NCT01908712) or rhLAMAN-09 (NCT01908725)]; participation in the CU programme vs follow-on trials depended on national regulations]. Patients who received placebo could switch to active treatment in the CU programme/follow-on studies. Long-term outcomes of VA treatment were assessed in comprehensive evaluation visits [last observation (LO)] undertaken per protocol for patients enrolled in rhLAMAN-07 or rhLAMAN-09 or as part of rhLAMAN-10 (open to patients receiving treatment within the CU programme) and were carried out in the same single centre. Data collected are presented here as an extension-phase analysis to the phase III trial.

### Patients

Patients with AM were screened for inclusion at Copenhagen University Hospital, Denmark, and enrolled in different European centres. Patients aged 5–35 years, with a confirmed diagnosis of AM as defined by alpha-mannosidase activity <10% of normal activity in blood leukocytes, and the ability to cooperate physically and mentally in trial assessments, were eligible for inclusion. Key exclusion criteria are listed in the “Supplementary” section.

### Procedures/treatment

All patients were evaluated for eligibility at the screening visit (Visit −1). Patients who provided informed consent completed a week of baseline assessments (Visit 0). Randomisation was stratified by age, and patients were allocated into blocks of five before randomisation in a 3:2 ratio to receive VA or placebo. Patients received IV infusion of VA 1 mg/kg or placebo as part of Visit 1 (first infusion) and once weekly thereafter (every seventh day ±3 days) for 52 weeks ±3 weeks. All efficacy endpoints were evaluated at Copenhagen University Hospital at weeks 26 and 52, with end-of-study visit at week 56. After completion of the phase III trial, patients either continued receiving active treatment, or switched from placebo to active treatment, as part of the CU programme/follow-on trials and contributed to assessment of long-term outcomes during follow-on trials or as part of rhLAMAN-10.

### Endpoints

The co-primary endpoints were change from baseline to week 52 in serum oligosaccharides and the 3-min stair climb test (3MSCT). The prioritised secondary endpoints were change from baseline to week 52 in the 6-min walk test (6MWT) and in forced vital capacity percentage (FVC %, measured by spirometry) of predicted normal value. Other secondary endpoints are detailed in the “Supplementary” section. Treatment-emergent adverse events (TEAEs) were assessed throughout the study and coded according to the* Medical Dictionary for Regulatory Activities* (MedDRA), version 16.0. Vital signs, physical examination results, clinical laboratory parameters and the development of VA antibodies were assessed throughout the study. Serum immunoglobulin G (IgG) levels were measured as part of the safety assessment. Endpoint hierarchy was maintained in the extension phase.

### Statistical analysis

No formal estimation of sample size was performed. The enrolment of 25 patients was deemed a good compromise between the limited availability of patients and the minimal amount of data required for assessment of efficacy and safety. Efficacy was defined as a statistically significant reduction in serum oligosaccharides (*P* < 0.025) and a trend for improvement in the 3MSCT and one prioritised secondary endpoint at the 52-week analysis. Early study completion was allowed at week 26 as per criteria reported in the “Supplementary” section.

Analysis of primary and prioritised secondary endpoints was performed on the relative change from baseline to week 52 in the full-analysis population. Data were log-transformed and submitted to an analysis of covariance (ANCOVA), with treatment as a fixed factor and corresponding baseline values and age as continuous covariates. Adjusted means in each treatment group, adjusted mean difference between VA and placebo, their 95% confidence intervals (CIs) and associated *P* values were estimated using this model. Additional information regarding statistical analysis and post hoc analyses are discussed in the “Supplementary” section.

For the extension-phase analysis, efficacy endpoints (actual values, absolute change and percentage change from baseline) were summarised for patients who received active treatment throughout the phase 3 study (VA arm) and placebo-group patients who switched to active treatment poststudy completion (placebo–VA switch arm). All analyses were descriptive only.

## Results

### Patients

Twenty-five European patients were enrolled and randomised to receive VA (*n* = 15) or placebo (*n* = 10). One additional patient was screened but not enrolled due to high IgE levels (Supplementary Fig. 1). Prespecified criteria for early discontinuation due to demonstrated efficacy were not reached at week 26. The study was continued as blinded up to the 52-week evaluation. Patient baseline characteristics are presented in Supplementary Table 1. All 25 patients completed the phase III study period and received VA treatment after study completion. Long-term follow-up data were available for 23 patients (Supplementary Fig. 1). Extension-phase analysis included outcomes after 12–18 months of treatment exposure for placebo-group patients who switched to active treatment, and 24–36 months for patients who received active treatment from the beginning of phase III. Compliance in the 12-month phase III trial was >90% for all 25 patients.

### Co-primary endpoints

**Fig. 1:**
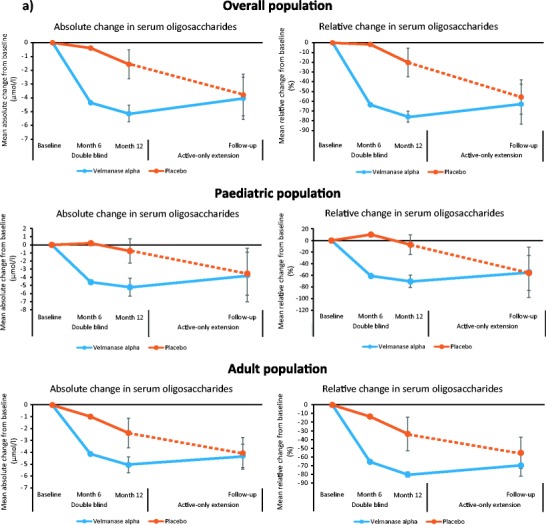

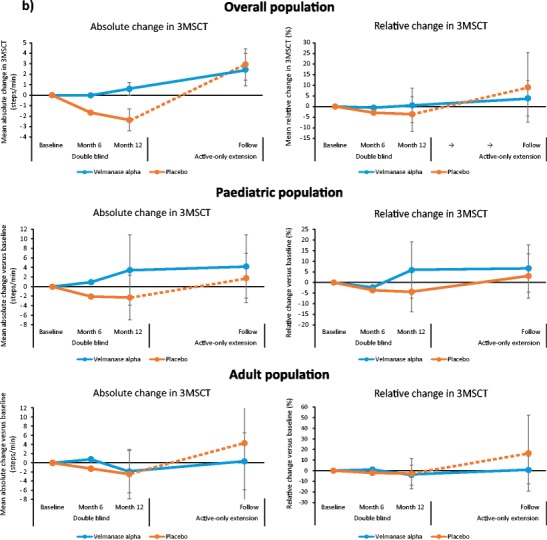
Mean absolute changes and mean relative changes in **a** serum oligosaccharides and **b** 3-min stair-climb test (3MSCT) from baseline to last observation

Descriptive analyses of changes in serum oligosaccharide levels by overall population and age subgroups from baseline to LO are shown in Fig. 1a and Supplementary Fig. 2a. Mean relative change in serum oligosaccharide concentration from baseline to week 52 was greater in patients receiving VA (*n* = 15; −77.6%; 95% CI:–81.6 to −72.8) vs placebo (*n* = 10; −24.1%; 95% CI:–40.3 to −3.6). The adjusted mean difference for VA vs placebo was −70.5% (95% CI –78.4 to −59.7; *P* < 0.001). At LO, the mean relative change from baseline was −62.9% (*n* = 13; 95% CI:–85.8 to −40.0) in the VA arm and − 55.7% (*n* = 9; 95% CI:–76.4 to –34.9) in the placebo–VA switch arm.

Descriptive analyses of absolute and relative changes in 3MSCT for the overall population and by age group from baseline to LO visit are shown in Fig. 1b and Supplementary Fig. 2b. Mean relative change in 3MSCT from baseline to week 52 was −1.1% (95% CI –9.0 to 7.6) with VA and − .0% (95% CI –13.4 to 6.5) with placebo [adjusted mean difference for VA vs placebo: +3.0% (95% CI –9.86 to 17.7; *P* = 0.648]]. At LO, mean relative change from baseline was +3.9% (*n* = 13; 95% CI –5.5 to 13.2) in the VA arm and + 9.0% (*n* = 9; 95% CI:–10.3 to 28.3) in the placebo–VA switch arm.

### Prioritised secondary efficacy endpoints

Data for prioritised secondary endpoints by overall population and age subgroups are shown in Table 1 and Supplementary Fig. 3. A slight increase in the 6MWT at week 52 was seen in the VA group compared with a slight decline in the placebo group (not statistically significant). The adjusted mean difference in relative change for VA vs placebo was 1.9% (95% CI –6.6 to 11.1; *P* = 0.66). At LO, the mean relative change was 0.7% (*n* = 13; 95% CI:–5.9 to 7.3) in the VA arm and 2.2% (*n* = 9; 95% CI:–7.8 to 12.3) in the placebo–VA switch arm.

**Table 1:** Summary of prioritised secondary endpoint results

	Mean change from baseline to week 52*		Mean change from baseline to last observation^b^	
	Velmanase alfa	Placebo	Velmanase alfa	Placebo
6MWT				
Overall population				
Absolute change, mean (95% CI)	*n* = 15 3.7 (−20.3 to 27.8)	*n* = 10 –3.6 (−33.1 to 25.9)	*n* = 13 2.8 (−28.3 to 34.0)	*n* = 9 3.2 (−36.0 to 42.5)
Percentage relative change (95% CI)	*n* = 15 0.6 (−4.74 to 6.32)	*n* = 10 –1.2 (−7.63 to 5.68)	*n* = 13 0.7 (−5.9 to 7.3)	*n* = 9 2.2 (−7.8 to 12.3)
Paediatric population				
Absolute change, mean (SD)	*n* = 7 12.3 (43.2)	*n* = 5 3.6 (43.0)	*n* = 7 24.6 (35.4)	*n* = 5 4.2 (41.9)
Percentage relative change (SD)	*n* = 7 2.0 (7.8)	*n* = 5 1.2 (9.4)	*n* = 7 5.3 (7.3)	*n* = 5 0.9 (8.6)
Adult population				
Absolute change, mean (SD)	*n* = 8 –2.5 (50.4)	*n* = 5–12.8 (41.6)	*n* = 6–22.5 (58.7)	*n* = 4 2.0 (67.8)
Percentage relative change (SD)	*n* = 8 0.4 (11.7)	*n* = 5–2.8 (12.8)	*n* = 6–4.7 (12.5)	*n* = 4 3.9 (18.7)
Forced vital capacity				
Overall population				
Absolute change, percentage of predicted (95% CI)	*n* = 15 8.2 (1.8 to 14.6)	*n* = 10 2.3 (−6.2 to 10.8)	*n* = 10 12.4 (2.3 to 22.5)	*n* = 8 4.0 (−13.7 to 21.7)
Percentage relative change (95% CI)	*n* = 15 10.1 (1.32 to 19.7)	*n* = 10 1.6 (−9.5 to 14.0)	–	–
Paediatric population				
Absolute change, percentage of predicted (SD)	*n* = 6 14.2 (8.7)	*n* = 4 8.0 (4.2)	*n* = 5 23.8 (10.1)	*n* = 4 10.0 (8.6)
Percentage relative change (SD)	20.5 (11.2)	9.5 (5.6)	–	–
Adult population				
Absolute change, percentage of predicted (SD)	*n* = 6 2.2 (7.2)	*n* = 5–2.8 (15.5)	*n* = 5 1.0 (4.74)	*n* = 4–2.0 (29.5)
Percentage relative change (SD)	2.3 (7.5)	−4.1 (18.7)	–	–

*6MWT* 6-min walking test,* CI* confidence interval,* FVC* forced vital capacity,* SD* standard deviation

^a^Data presented in overall population are adjusted mean changes (95% CI) derived from analysis of covariance analyses; data for the paediatric and adult populations were post hoc analyses and are summarised as mean (SD)

^b^Baseline to last observation analyses are descriptive only

Change from baseline of FVC % resulted numerically in favour (not statistically significant) of VA compared with placebo at week 52. The adjusted mean difference in relative change for VA vs placebo was 8.4% (95% CI –6.1 to 25.1; *P* = 0.27). At LO, mean relative change was 12.4% (*n* = 10; 95% CI 2.3 to 22.5) in the VA arm and 4.0 (*n* = 8; 95% CI:–13.7 to 21.7) in the placebo–VA switch arm. The results of additional secondary endpoints including audiometry, cerebrospinal fluid biomarkers and use of help and aids are presented in Supplementary Tables 2–6.

**Fig. 2:**
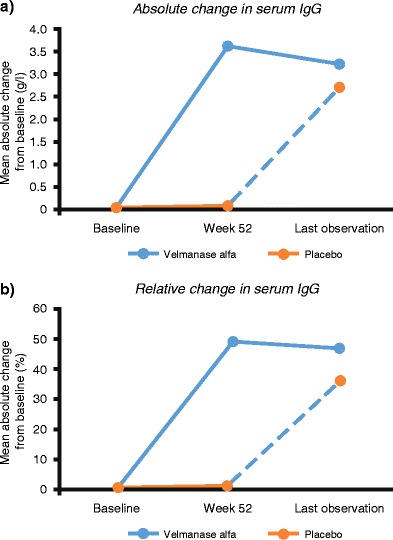
Serum immunoglobulin changes in the overall population as measured by **a** mean absolute change and **b** mean relative change

Post hoc analysis of serum IgG showed a significant difference in mean absolute change from baseline to week 52 with VA treatment compared with placebo (Fig. 2) [between-group difference for VA vs placebo: 3.5 g/l (95% CI 2.1 to 4.8); *P* < 0.001]. At LO, mean relative change from baseline in serum IgG was 47.2% (*n* = 14; 95% CI:30.2 to 64.3) in the VA arm and 37.3% (*n* = 9; 95% CI:24.9 to 49.7) in the placebo–VA switch arm.

### Safety

TEAEs are summarised in Table 2. Five serious TEAEs occurred with VA treatment, of which four were considered as unrelated to treatment. One case of moderate, acute renal failure considered possibly related to treatment occurred after almost 12 months of treatment in a patient known to be receiving long-term concomitant ibuprofen. VA was temporarily suspended, and the patient recovered after 92 days. The patient restarted active treatment with no safety issues reported and subsequently enrolled into the CU programme. No TEAEs led to discontinuation from the study, and no deaths were reported.

**Table 2:** Summary of treatment-emergent adverse events during the 12-month phase III study period

	Velmanase alfa *n* = 15		Placebo *n* = 10	
	No. of patients (%)	Events	No. of patients (%)	Events
Any TEAEs	15 (100)	157	9 (90.0)	113
Treatment-related TEAEs^a^	7 (46.7)	30	5 (50.0)	9
Serious TEAEs^b^	5 (33.3)	5	0 (0.0)	0
Serious treatment-related TEAEs	1 (6.7)	1	0 (0.0)	0
Severe TEAEs	1 (6.7)	1	0 (0.0)	0
TEAEs with a fatal outcome	0 (0.0)	0	0 (0.0)	0
TEAEs leading to discontinuation	0 (0.0)	0	0 (0.0)	0

*TEAEs* treatment-emergent adverse events

^a^Treatment-related TEAEs were defined as having occurred in a reasonable temporal sequence from the time of study-drug administration, abated upon discontinuation of the study drug and reappeared when treatment with the study drug was restarted

^b^Serious TEAEs were defined as resulting in one of the following: death, life-threatening experience, a requirement for/prolongation of hospitalisation, a persistent or significant disability/incapacity, a congenital anomaly/birth defect or an important medical event that jeopardised the patient or subject and may require medical or surgical intervention to prevent one of the outcomes listed above

Eight patients (three in the VA group and five in the placebo arm) tested positive for anti-VA IgG antibodies (ADA+) on at least one occasion before treatment (active or placebo); the three patients of the VA arm and two of the placebo switch group had one positive results during on-treatment assessments. All patients presented ADA levels around the set cutoff level; just one patient receiving VA had ADA > 80 U/ml and experienced infusion-related reactions (IRRs).

## Discussion

In this phase III trial, VA was associated with a marked and statistically significant clearance of serum oligosaccharides vs placebo in patients with AM. This improvement in serum oligosaccharide clearance continued into the extension phase. Marginal clearance of oligosaccharides was also observed in four patients of the placebo–VA switch arm, although this was numerically smaller than in patients initially randomised to active treatment; this is considered mainly due to spontaneous fluctuations in oligosaccharide concentration at the 52-week visit. Reducing the accumulation of mannosyl-rich oligosaccharides in AM patients is assumed to target the root cause of the systemic disease, since such accumulation is central to the disease pathology and is considered related to cellular dysfunction (Malm et al. 2014). Due to the lack of power, no statistically significant results were detected vs placebo in the motor and pulmonary function endpoints, but the changes observed with VA treatment in the overall population resulted consistently in the direction of a trend of improvement, especially in paediatric patients.

The combination of 3MSCT and 6MWT provides a robust approach for evaluating the impact of ERT on patient endurance. Stair climbing requires greater muscle strength and range of joint motion than level walking and is therefore better able to demonstrate functional difficulties (Nightingale et al. 2014). There was a marginal improvement in the 3MSCT for VA vs placebo group. It should be noted that the placebo group had better baseline functional status (40.0% patients accomplished ≥65 steps/min in the 3MSCT and ≥ 500 m in the 6MWT vs 13.3% in the VA group), suggesting that the population randomised to VA had greater motor limitation than those in the placebo group.

Post hoc analysis revealed a better result (but not statistically significant) of the 3MSCT in paediatric patients receiving VA compared with adults, suggesting that VA produces greater clinical benefits in motor function when administered early in the disease course. Similar findings linking early treatment with better outcomes have been reported in other ERT studies (Muenzer 2014; Gabrielli et al. 2010; Tylki-Szymanska et al. 2012; Tajima et al. 2013). In the extension phase, looking at patient-specific results, greater improvements in 3MSCT can be observed in patients in the placebo–VA switch arm compared with patients initially randomised to the active treatment arm. Two of the four adult patients who switched from placebo to treatment showed an increase in 3MSCT at LO above their initial baseline after declining during 12 months of placebo treatment. One additional switch patient who improved by 7.7 steps/min during the placebo phase further improved by 15 steps/min in the first 12–18 months after starting treatment. These patient-specific results are consistent with a stabilisation (and in some cases improvement) of motor endurance, even in adults.

At baseline, the paediatric subgroup reported values of FVC compatible with a pulmonary restrictive syndrome (Keddissi et al. 2013) but reverted to normal pulmonary function during the trial. These results suggest VA might prevent worsening pulmonary function and may correct pulmonary dysfunction in paediatric patients. The smaller improvements observed in adults could be related to high baseline values creating a ceiling effect or may indicate stabilisation in pulmonary function decline.

It is speculated that high levels of circulating oligosaccharides (in addition to intracellular accumulation) may contribute to the immunodeficiency seen in AM patients, since they bind to interleukin-2 (IL-2) receptors, disturbing IL-2-dependent responses (Zanetta et al. 1998). Post hoc analyses revealed a significant increase in serum IgG levels with VA vs placebo, and an increase in levels in the placebo–VA switch arm during the extension phase, suggesting that serum IgG levels could be a potential biomarker of positive treatment activity and response. Immunodeficiency is a major cause of recurrent infections in AM patients and can lead to early death; therefore, increased IgG levels may be relevant in AM and have a therapeutic benefit. A postmarketing registry study will help in understanding whether increased IgG correlates with reduction of infection rates.

VA 1 mg/kg administered once weekly was well tolerated. ADAs were detected in a limited number of participants, suggestive of a clear and reproducible pharmacological effect with VA. Only one patient developed a manifestly positive ADA level and experienced IRR. The patient received premedication prior to subsequent infusions and continued to benefit from treatment with regard to motor function, suggesting that IRRs may be a manageable aspect of treatment. At LO, the serum oligosaccharides presented as high as at baseline, with persistence of high ADA levels.

The significant clearance of serum oligosaccharides in this study is consistent with the findings of the phase II study (Borgwardt et al. 2013). However, 3MSCT results did not reach the same level of clinical improvement observed at phase II. This may be accounted for by differences in the patient population between the two studies. The entirely paediatric phase II study population performed better at baseline in the 3MSCT and 6MWT compared with the phase III population, suggesting that clinical severity was worse in phase III patients. Post hoc analyses have investigated different baseline characteristics, such as age, gender, genotype, residual enzymatic activity and disease burden measured by Childhood Health Assessment Questionnaire (CHAQ) Disability Index (DI). No baseline factor was found to be predictive of VA treatment outcome besides age (data on file). Future postmarketing data collection might shed light on possible baseline characteristic that might predict treatment outcome, but based on data currently available and the published medical literature on the disease, no conclusive observations can be made.

The efficacy endpoints in this study were assessed by the same personnel at one central site, thus increasing the reliability of collected results. However, there are limitations of the study design; notably, the study population was small, which is inevitable given the rarity of the disease, and it was also heterogeneous and stratified by age but not by baseline disability. This led to imbalanced baseline endurance ability allocation, which was poorer in the VA arm. Moreover, primary and key secondary endpoints are to some extent dependent on patient collaboration and understanding, enhancing the variability of results.

This study demonstrates the significant biological activity of VA in reducing serum oligosaccharide levels and increasing serum IgG across all ages. Positive changes in endurance and pulmonary function with VA, particularly in paediatric patients with subnormal respiratory function, suggests that greater clinical benefits could be obtained if patients are diagnosed and begin treatment early in the disease course (< 18 years). VA was well tolerated, with no significant safety or immunogenicity concerns raised. Comprehensive long-term data of the full clinical study programme have been evaluated in the rhLAMAN-10 study and integrated analysis (Lund et al. 2018; NCT02478840).

## Electronic supplementary material

ESM 1 (DOCX 15 kb)

Supplementary Table 1 Baseline characteristics (DOCX 17 kb)

Supplementary Table 2 Summary of additional secondary endpoints (DOCX 13 kb)

Supplementary Table 3 Pure Tone Audiometry (DOCX 12 kb)

Supplementary Table 4 Summary of central nervous system (CNS) biomarker results (DOCX 13 kb)

Supplementary Table 5 Use of help and aids matrix table: baseline vs month 12 (DOCX 12 kb)

Supplementary Table 6 Use of help and aids matrix table: baseline vs extension-phase follow-up (DOCX 13 kb)

Supplementary Fig. 1 Consolidated Standards of Reporting Trials (CONSORT) diagram (JPEG 883 kb)

Supplementary Figure 2 Individual absolute change at month 12 and at last observation versus baseline in** a** serum oligosaccharides and** b** 3-min stair-climb test (3MSCT) (JPEG 126 kb)

Supplementary Fig. 3 Individual absolute change at month 12 and at last observation versus baseline in** a** 6-min walk test (6MWT) and** b** forced vital capacity (FVC) % (JPEG 119 kb)
