# Supplementary material for: Efficacy and safety of Velmanase alfa in the treatment of patients with alpha-mannosidosis: results from the core and extension phase analysis of a phase III multicentre, double-blind, randomised, placebo-controlled trial
Source: J Inherit Metab Dis. 2018 May 30;41(6):1215–23. doi: 10.1007/s10545-018-0185-0 (PMC6326984; doi:10.1007/s10545-018-0185-0)
Supplement: Supplementary file 1 — (DOCX 15 kb) [file 10545_2018_185_MOESM1_ESM.docx]

**Supplementary methods**

**Key exclusion criteria**

Presence of known chromosomal abnormality and syndromes, other than AM; inability to walk without support (the use of walking aids/wheelchair for partial support and longer distances was permitted); history of bone marrow transplantation; any psychotic disease, active or in remission; or total immunoglobulin E (IgE) >800 IU/ml. Eligibility was not limited by motor performance at baseline.

**Criteria for early study completion**

The protocol allowed for early study completion based on successful demonstration of efficacy at week 26 (statistically significant reduction in serum oligosaccharides and a statistically significant improvement in the 3-minute stair climb test (3MSCT) under significance levels of *P* < 0.025 and *P* < 0.05, respectively).

**Additional information regarding statistical analysis and post hoc analyses**

The same analysis of covariance (ANCOVA) model used for the primary analysis was applied on absolute change from baseline to weeks 26 and 52 and on the log-transformed relative change from baseline to week 26. A responder analysis was also undertaken. For CHAQ (Disability Index and VAS pain) and EQ5D5L assessments, actual values and changes from baseline were summarised at each visit by treatment group using descriptive statistics.

The analysis of all safety variables was performed in the safety population, defined as all randomised patients who received at least one dose of the study treatment.

Post hoc analyses were used to evaluate co-primary and prioritised secondary endpoints, Bruininks-Oseretsky Test of Motor Proficiency (BOT-2) and pulmonary function tests by age subgroups (paediatric [<18 years] vs adult [≥18 years]) using descriptive statistics. A further post hoc analysis was conducted to evaluate absolute changes from baseline to week 52 in serum immunoglobulin, using an ANCOVA model with treatment as a fixed factor and baseline value and age as continuous covariates.
