# Supplementary material for: Efficacy and safety of Velmanase alfa in the treatment of patients with alpha-mannosidosis: results from the core and extension phase analysis of a phase III multicentre, double-blind, randomised, placebo-controlled trial
Source: J Inherit Metab Dis. 2018 May 30;41(6):1215–23. doi: 10.1007/s10545-018-0185-0 (PMC6326984; doi:10.1007/s10545-018-0185-0)
Supplement: Supplementary file 2 — Baseline characteristics (DOCX 17 kb) [file 10545_2018_185_MOESM2_ESM.docx]

|  | | | | | | | | | |
| --- | --- | --- | --- | --- | --- | --- | --- | --- | --- |
| **#** | | **Treatment** | **Sex** | **Age class at 1st dose** | **Genotype** | **Oligosaccharides (umol/L)** | **3MSCT(step/min)** | **6MWT (m)** | **FVC(% of predicted)** |
| 001 | | Velmanase alfa | F | >= 18 years | Genotype Group 1 | 7.5 | 45.3 | 430 | 96 |
| 002 | | Placebo | M | >= 18 years | Genotype Group 2 | 6.3 | 38.3 | 219 | 85 |
| 003 | | Velmanase alfa | M | >= 18 years | Genotype Group 2 | 7 | 45.0 | 422 | 97 |
| 004 | | Velmanase alfa | M | >= 18 years | Genotype Group 1 | 5 | 56.7 | 490 |  |
| 005 | | Placebo | F | >= 18 years | Genotype Group 3 | 7.1 | 32.0 | 319 | 92 |
| 006 | | Placebo | M | >= 18 years | Genotype Group 3 | 8.4 | 70.3 | 594 | 95 |
| 007 | | Velmanase alfa | M | >= 18 years | Genotype Group 2 | 7.8 | 50.0 | 480 | 119 |
| 008 | | Velmanase alfa | F | >= 18 years | Genotype Group 2 | 5.6 | 48.0 | 452 | 75 |
| 009 | | Velmanase alfa | F | >= 18 years | Genotype Group 2 | 6.1 | 37.7 | 335 | 72 |
| 010 | | Placebo | F | >= 18 years | Genotype Group 2 | 8.2 | 47.3 | 485 | 81 |
| 011 | | Velmanase alfa | M | >= 18 years | Genotype Group 3 | 6.4 | 47.3 | 491 |  |
| 012 | | Placebo | M | >= 18 years | Genotype Group 2 | 6.2 | 78.0 | 696 | 109 |
| 013 | | Velmanase alfa | M | >= 18 years | Genotype Group 2 | 4.9 | 70.3 | 627 | 103 |
| 014 | | Velmanase alfa | F | 12 \|-18 years | Genotype Group 1 | 7.1 | 48.0 | 422 | 80 |
| 015 | | Placebo | F | 12 \|-18 years | Genotype Group 1 | 10.2 | 72.3 | 543 | 93 |
| 016 | | Placebo | F | 12 \|-18 years | Genotype Group 3 | 5.7 | 49.0 | 480 | 96 |
| 017 | | Placebo | F | 12 \|-18 years | Genotype Group 2 | 4.4 | 66.3 | 510 | 72 |
| 018 | | Velmanase alfa | M | 12 \|-18 years | Genotype Group 1 | 7.9 | 45.3 | 480 | 76 |
| 019 | | Velmanase alfa | M | 12 \|-18 years | Genotype Group 2 | 6 | 83.3 | 586 | 94 |
| 020 | | Velmanase alfa | F | <12 years | Genotype Group 1 | 8.1 | 55.0 | 434 | 50 |
| 021 | | Velmanase alfa | M | <12 years | Genotype Group 2 | 5.9 | 55.0 | 420 | 54 |
| 022 | | Placebo | M | <12 years | Genotype Group 2 | 5.3 | 60.0 | 476 | 91 |
| 023 | | Velmanase alfa | M | <12 years | Genotype Group 2 | 8.7 | 53.7 | 427 | 64 |
| 024 | | Placebo | M | <12 years | Genotype Group 1 | 4.5 | 41.3 | 335 |  |
| 025 | | Velmanase alfa | F | <12 years | Genotype Group 2 | 7.7 | 53.3 | 398 |  |
