# Supplementary material for: Efficacy and safety of Velmanase alfa in the treatment of patients with alpha-mannosidosis: results from the core and extension phase analysis of a phase III multicentre, double-blind, randomised, placebo-controlled trial
Source: J Inherit Metab Dis. 2018 May 30;41(6):1215–23. doi: 10.1007/s10545-018-0185-0 (PMC6326984; doi:10.1007/s10545-018-0185-0)
Supplement: Supplementary file 3 — Summary of additional secondary endpoints (DOCX 13 kb) [file 10545_2018_185_MOESM3_ESM.docx]

**Supplementary Table 2** Summary of additional secondary endpoints

|  | **Mean change from baseline to week 52** | |
| --- | --- | --- |
|  | **Velmanase alfa**  ***n* = 15** | **Placebo**  ***n* = 10** |
| **Total Equivalence Age (Leiter-AM)** | | |
| Absolute change, years (SD) | 0.0 (1.4) | 0.1 (1.0) |
| Percentage relative change (SD) | 5.6 (23.0) | 3.8 (14.6) |
| **Total Equivalence Age (Leiter-VR)** | | |
| Absolute change, years (SD) | 0.2 (0.7) | 0.2 (0.7) |
| Percentage relative change (SD) | 5.6 (13.7) | 3.3 (8.2) |
| **BOT-2 Total Score** | | |
| Absolute change, points (SD) | 6.4 (13.4) | –0.3 (9.6) |
| Percentage relative change (SD) | 12.3 (20.6) | 3.5 (14.2) |
| **CHAQ Disability Index** | | |
| Absolute change, score (SD) | –0.0 (0.3) | 0.2 (0.4) |
| Percentage relative change (SD) | – | – |
| **CHAQ VAS Pain** | | |
| Absolute change, score (SD) | 0.2 (0.7) | 0.2 (0.7) |
| Percentage relative change (SD) | – | – |
| **EQ5D5L Index** | | |
| Absolute change, score (SD) | 0.0 (0.1) | 0.03 (0.2) |
| Percentage relative change (SD) | – | – |

AM, Attention and Memory; BOT-2, Bruininks-Oseretsky Test of Motor Proficiency; CHAQ, Childhood Health Assessment Questionnaire; SD, standard deviation; VR, Visualization and Reasoning
