# Supplementary material for: Efficacy and safety of Velmanase alfa in the treatment of patients with alpha-mannosidosis: results from the core and extension phase analysis of a phase III multicentre, double-blind, randomised, placebo-controlled trial
Source: J Inherit Metab Dis. 2018 May 30;41(6):1215–23. doi: 10.1007/s10545-018-0185-0 (PMC6326984; doi:10.1007/s10545-018-0185-0)
Supplement: Supplementary file 4 — Pure Tone Audiometry (DOCX 12 kb) [file 10545_2018_185_MOESM4_ESM.docx]

**Supplementary Table 3** Pure Tone Audiometry

|  | **Mean change from baseline to 52** | |  |
| --- | --- | --- | --- |
|  | **Velmanase alfa** | **Placebo** |  |
| **Bone conduction (best ear)** | | | |
| N | 14 | 10 |  |
| Absolute change, dB (SD) | 2.4 (5.2) | 0.1 (5.9) |  |
| Percentage relative change (SD) | 6.2 (13.7) | –0.7 (10.8) |  |
| **Air conduction (left ear)** | | | |
| N | 15 | 10 |  |
| Absolute change, dB (SD) | 1.0 (8.0) | 0.8 (7.8) |  |
| Percentage relative change (SD) | 3.3 (14.3) | 3.0 (16.5) |  |
| **Air conduction (right ear)** | | | |
| N | 15 | 10 |  |
| Absolute change, dB (SD) | 1.9 (11.3) | –1.9 (9.0) |  |
| Percentage relative change (SD) | 4.9 (17.4) | –2.8 (14.6) |  |

dB, decibel; SD, standard deviation
