# Supplementary material for: Efficacy and safety of Velmanase alfa in the treatment of patients with alpha-mannosidosis: results from the core and extension phase analysis of a phase III multicentre, double-blind, randomised, placebo-controlled trial
Source: J Inherit Metab Dis. 2018 May 30;41(6):1215–23. doi: 10.1007/s10545-018-0185-0 (PMC6326984; doi:10.1007/s10545-018-0185-0)
Supplement: Supplementary file 5 — Summary of central nervous system (CNS) biomarker results (DOCX 13 kb) [file 10545_2018_185_MOESM5_ESM.docx]

**Supplementary Table 4** Summary of CNS biomarkers results

|  | **Mean change from baseline to week 52** | |
| --- | --- | --- |
|  | **Velmanase alfa**  ***n* = 15** | **Placebo**  ***n* = 10** |
| **CSF-oligosaccharides** | | |
| Absolute change, μmol/l (SD) | –0.4 (1.2) | –0.5 (0.9) |
| (SD) | –4.3 (10.6) | –5.0 (8.0) |
| **CSF-Tau** | | |
| Absolute change, ng/l (SD) | –61.0 (117.5) | –60.1 (70.2) |
| Percentage relative change (SD) | –5.2 (14.9) | –10.5 (13.3) |
| **CSF-NFL** | | |
| Absolute change, ng/l (SD) | –15.3 (87.2) | –112.0 (598.0) |
| Percentage relative change (SD) | –1.4 (20.6) | 4.3 (44.5) |
| **CSF-GFAp** | | |
| Absolute change, ng/l (SD) | 107.3 (187.6) | 141.0 (231.9) |
| Percentage relative change (SD) | 66.1 (67.5) | 40.3 (50.3) |

CNS, central nervous system; CSF, cerebrospinal fluid; SD, standard deviation
