# Supplementary material for: Efficacy and safety of Velmanase alfa in the treatment of patients with alpha-mannosidosis: results from the core and extension phase analysis of a phase III multicentre, double-blind, randomised, placebo-controlled trial
Source: J Inherit Metab Dis. 2018 May 30;41(6):1215–23. doi: 10.1007/s10545-018-0185-0 (PMC6326984; doi:10.1007/s10545-018-0185-0)
Supplement: Supplementary file 6 — Use of help and aids matrix table: baseline vs month 12 (DOCX 12 kb) [file 10545_2018_185_MOESM6_ESM.docx]

**Supplementary Table 5** Use of help and aids matrix table: baseline vs month 12

|  | | **Requires help and aids at baseline** | | |
| --- | --- | --- | --- | --- |
|  |  | No | Yes | Total |
| **Requires help and aids at month 12** | Velmanase alfa | | | |
|  | No | 8 | 2 | 10 |
|  | Yes | 2 | 3 | 5 |
|  | Total | 10 | 5 | 15 |
|  | Placebo | | | |
|  | No | 3 | 2 | 5 |
|  | Yes | 2 | 3 | 5 |
|  | Total | 5 | 5 | 10 |
|  | Total | | | |
|  | No | 11 | 4 | 15 |
|  | Yes | 4 | 6 | 10 |
|  | Total | 15 | 10 | 25 |
