# Supplementary material for: Efficacy and safety of Velmanase alfa in the treatment of patients with alpha-mannosidosis: results from the core and extension phase analysis of a phase III multicentre, double-blind, randomised, placebo-controlled trial
Source: J Inherit Metab Dis. 2018 May 30;41(6):1215–23. doi: 10.1007/s10545-018-0185-0 (PMC6326984; doi:10.1007/s10545-018-0185-0)
Supplement: Supplementary file 7 — Use of help and aids matrix table: baseline vs extension-phase follow-up (DOCX 13 kb) [file 10545_2018_185_MOESM7_ESM.docx]

**Supplementary Table 6.** Use of help and aids matrix table: baseline vs last observation

|  | | **Requires help and aids at baseline** | | |
| --- | --- | --- | --- | --- |
|  |  | No | Yes | Total |
| **Requires help and aids at last observation** | Velmanase alfa | | | |
|  | No | 5 | 2 | 7 |
|  | Yes | 1 | 2 | 3 |
|  | Total | 6 | 4 | 10 |
|  | Placebo | | | |
|  | No | 2 | 4 | 6 |
|  | Yes | 1 | 0 | 1 |
|  | Total | 3 | 4 | 7 |
|  | Total | | | |
|  | No | 7 | 6 | 13 |
|  | Yes | 2 | 2 | 4 |
|  | Total | 9 | 8 | 17 |
