# Supplementary figures and images for: Efficacy and safety of Velmanase alfa in the treatment of patients with alpha-mannosidosis: results from the core and extension phase analysis of a phase III multicentre, double-blind, randomised, placebo-controlled trial
Source: J Inherit Metab Dis. 2018 May 30;41(6):1215–23. doi: 10.1007/s10545-018-0185-0 (PMC6326984; doi:10.1007/s10545-018-0185-0)

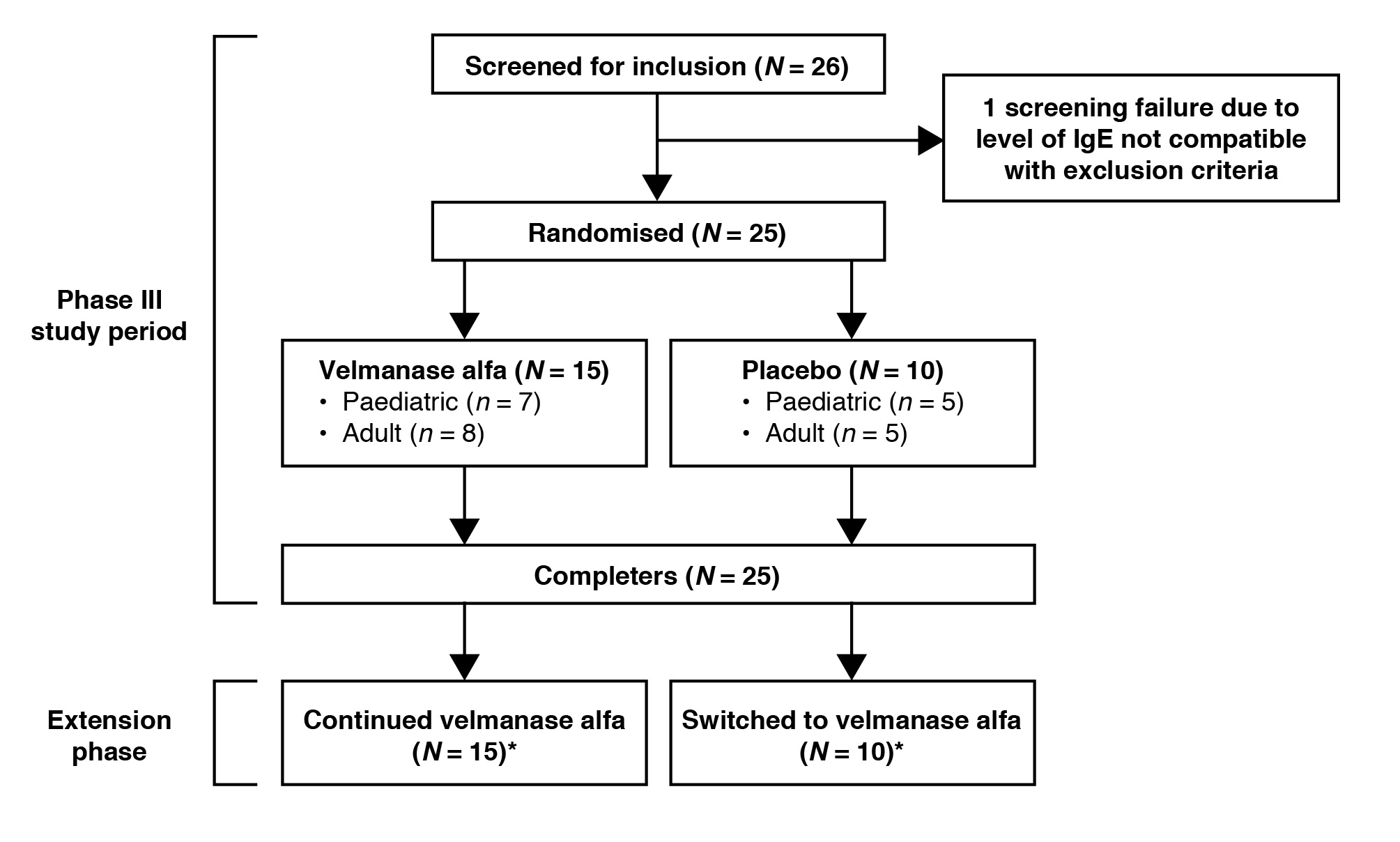

Supplement: Supplementary file 8 — Consolidated Standards of Reporting Trials (CONSORT) diagram (JPEG 883 kb) [file 10545_2018_185_MOESM8_ESM.jpg]

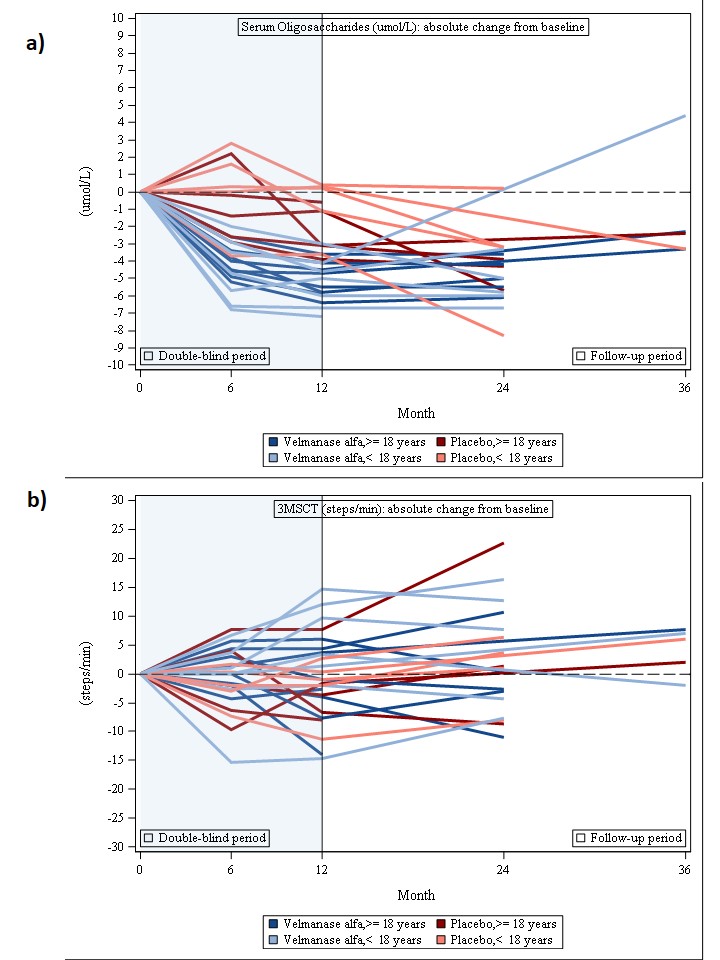

Supplement: Supplementary file 9 — Individual absolute change at month 12 and at last observation versus baseline in a serum oligosaccharides and b 3-min stair-climb test (3MSCT) (JPEG 126 kb) [file 10545_2018_185_MOESM9_ESM.jpg]

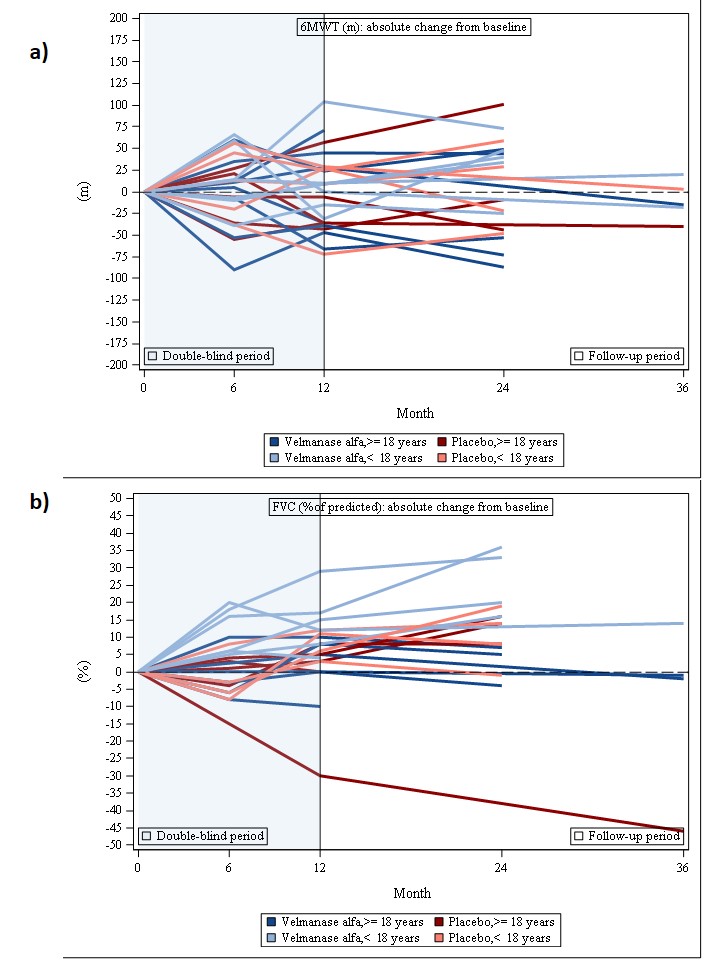

Supplement: Supplementary file 10 — Individual absolute change at month 12 and at last observation versus baseline in a 6-min walk test (6MWT) and b forced vital capacity (FVC) % (JPEG 119 kb) [file 10545_2018_185_MOESM10_ESM.jpg]
